# On the Variability and Increasing Trends of Heat Waves over India

**DOI:** 10.1038/srep26153

**Published:** 2016-05-19

**Authors:** P. Rohini, M. Rajeevan, A. K. Srivastava

**Affiliations:** 1 Indian Institute of Tropical Meteorology, Pune, 411 008, India; 2 India Meteorological Department, Pune, 411 005, India

## Abstract

Over India, heat waves occur during the summer months of April to June. A gridded daily temperature data set for the period, 1961–2013 has been analyzed to examine the variability and trends in heat waves over India. For identifying heat waves, the Excess Heat Factor (EHF) and 90^th^ percentile of maximum temperatures were used. Over central and northwestern parts of the country, frequency, total duration and maximum duration of heat waves are increasing. Anomalous persistent high with anti-cyclonic flow, supplemented with clear skies and depleted soil moisture are primarily responsible for the occurrence of heat waves over India. Variability of heat waves over India is influenced by both the tropical Indian Ocean and central Pacific SST anomalies. The warming of the tropical Indian Ocean and more frequent El Nino events in future may further lead to more frequent and longer lasting heat waves over India.

Due to increase in anthropogenic activities, global temperatures have shown a warming trend of 0.85 °C over the period 1880–2012 1. Annual surface air temperatures over India also have shown increasing trends of similar magnitude^2^ during the period 1901–2014. During the recent years, minimum temperatures (nighttime) have increased more than daytime temperatures, suggesting the possible role of moisture and the greenhouse gases^2^. The changes in mean value or variance in temperatures may cause increase in extreme temperatures and therefore occurrence of heat waves^1^,^3^. Heat waves are anomalous episodes with extremely high surface air temperatures, lasting for several days with serious consequences. Climate extremes like heat waves are of great interest globally and regionally due to their high impacts on various sectors including health, agriculture, ecosystems and national economy^4^,^5^,^6^,^7^,^8^,^9^,^10^,^11^,^12^,^13^,^14^. The health impacts of heat waves typically involve dehydration, heat cramps, heat exhaustion and/or heat stroke. Over India, other impacts due to heat waves could be agricultural crop failures and power outages due to excess consumption.

There has been a number of mega heatwaves in recent years worldwide. Recent studies suggest that frequency, duration and intensity of heat wave events are increasing over land regions across the globe^7^,^8^,^9^,^10^. Over India, in the past, heat waves have caused more deaths than any other natural hazard^15^. In May 2015, a severe heat wave affected parts of southeastern parts of India (Telangana and Andhra Pradesh) which claimed lives of more than 2500 people. This was an unprecedented event caused due to persisting anomalous atmospheric conditions due to delayed onset of southwest monsoon. Even though heat waves are important in many aspects, there are not many studies addressing heat waves over India except the recent paper by Pai *et al.*^10^. One serious constraint was the non-availability of quality controlled daily temperature data over the region. The study by Pai *et al.*^10^ considered temperature data of only 103 stations spread over the country.

In the present study, using a high resolution gridded daily temperature data set, we show that frequency, total duration of heat waves per season and maximum duration of heat waves are increasing over India during the summer season (April to June). This present study was possible due to the availability of high resolution gridded daily temperature data for the period 1961–2013. There is no universal method or criterion to monitor and classify heat waves globally or regionally. Out of many known indices, two indices, Excessive Heat Factor (EHF)^32^,^33^ and 90^th^ Percentile^32^,^33^ of daily maximum (daytime) temperatures were considered for estimating statistics of heat waves. The details of temperature data sets and the heat wave indices used in the study are discussed in the Methods section.

## Results

**Figure 1:**
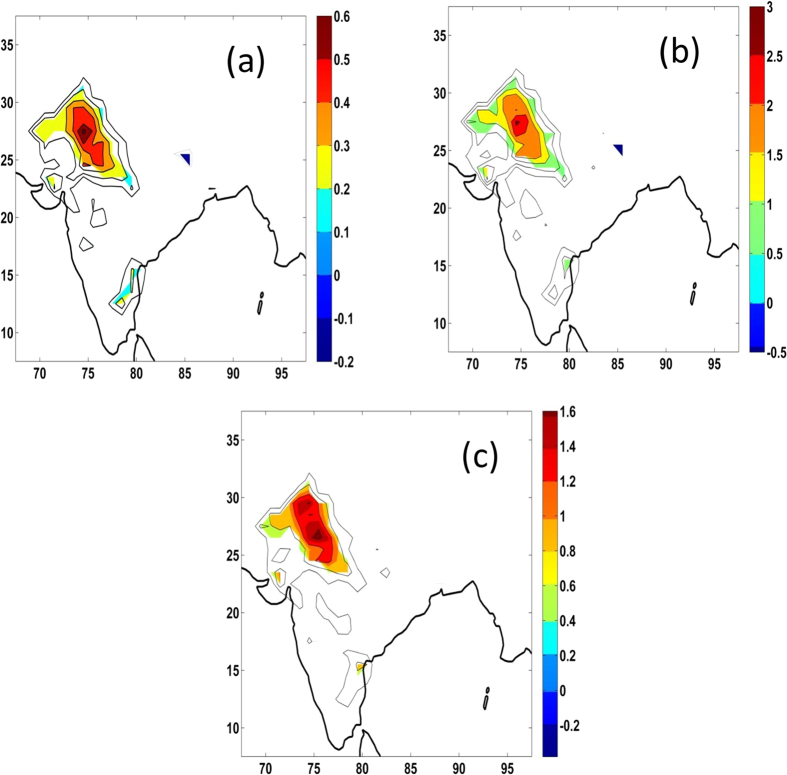
Trends in (**a**) frequency (**b**) total duration (days) and (**c**) maximum length of duration (days) of heat waves over India during the period 1961–2013. The trends which are statistically significant at the 95% level are only shown in colour shades. Statistical significance test was made using the Mann-Kendal test. This figure was prepared using the Matlab version R2012a software (http://in.mathworks.com).

Heat wave events occur over most of the country during April-May-June months, except along the west coast and northeast India (Fig. S1). There are two prominent areas of heat wave occurrence, one over northwest India and another over southeastern parts of India. On an average, one heat wave event occurs over northwest India with the total duration of about 5 days per season. Some heat wave events may last for more than 7 days. In a few years, no heat wave was reported. However, as shown in Fig. 1, there is a systematic increase in frequency, total duration and maximum duration of heat waves. Figure 1 shows the spatial distribution of linear trends of heat waves (frequency, average duration and maximum duration) during the period 1961–2013. The increasing trends of frequency, average duration and maximum duration are statistically significant (at the 95% confidence level) over northwest India and southeast India. The trends elsewhere are not statistically significant. Over northwest India, frequency of heat waves increased by 1 event in 20 years. Average total duration of heat waves increased by 2 days per decade, while maximum duration increased by about 1.5 days per decade. The trends over southeast India are not appreciable compared to the trends over northwest India. The present results are similar with the results documented by Pai *et al.*^10^, which used different criteria for heat waves and lesser number of stations. Pai *et al.*^10^ have documented significant increasing trends of severe heat waves over central and northwest India.

**Figure 2:**
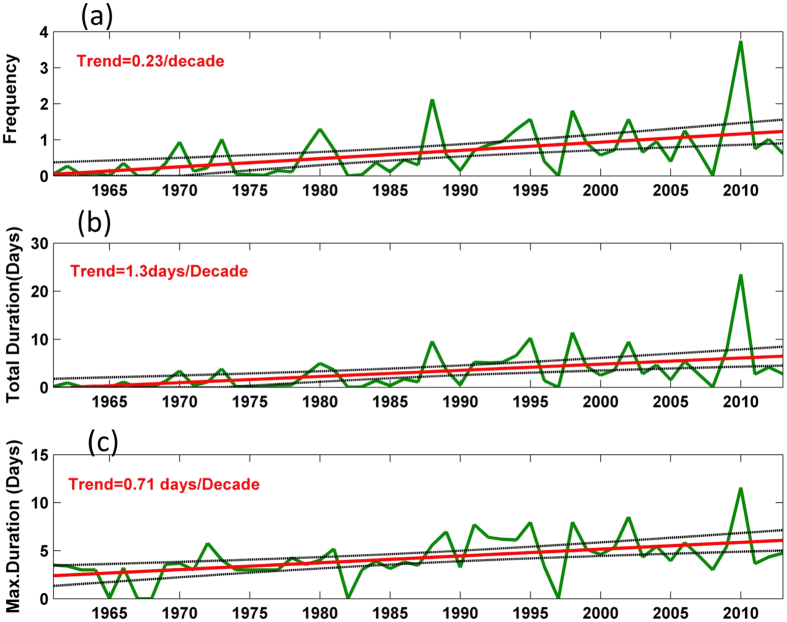
Time series of (**a**) frequency (**b**) total duration (days) and (**c**) maximum duration (days) of heat waves averaged over the region 22°–31°N, 70°–77°E. The linear trend line (red line) and the 95% confidence limits (black lines) are also shown. All the time series show statistically significant trends at the 95% confidence level. The magnitude of trends is shown on the upper left corner.

Time series of frequency, total duration and maximum duration of heat waves averaged over northwest India (22°N–31°N, 70°E–77°E) are shown in Fig. 2. The trend line and the 95% confidence levels are also indicated. All the time series are showing statistically significant (at the 95% confidence level) increasing trends. Frequency of heat waves averaged over northwest India has shown an increase of 0.23 per decade. Total duration of heat waves has shown an increase of 1.3 days per decade and maximum duration, 0.76 days per decade. The same time series based on 90^th^ percentile of maximum temperature (Tmax_90_) are shown in Fig. S2. The magnitude of trends based on Tmax_90_ are however smaller than the trends based on EHF. This could be due to the fact that EHF is based on the additional influence of nighttime temperatures, while Tmax_90_ is based on only daytime temperatures. Over India, nighttime temperatures have shown faster increasing trend compared to daytime temperatures^2^.

Heat waves are basically meteorological events, even though they are studied from a climatological perspective. Most of heat waves persist around one week. The development of heat wave is caused by synoptic environment and interaction of large-scale and small-scale processes. The previous studies^13^,^16^,^17^,^18^,^19^ have shown that heat waves across Europe and the United States are typically associated with atmospheric blocking events and large scale climate mode variability. Oceanic conditions also may contribute to heat wave development^17^. In case of the 2003 European heat wave, large scale blocking circulation enabled clear skies with an increasingly dry land surface resulting in exceptionally high temperatures. For Australian heat waves, mid-latitude baroclinic wave trains have been identified as a dominant mechanism^19^.

**Figure 3:**
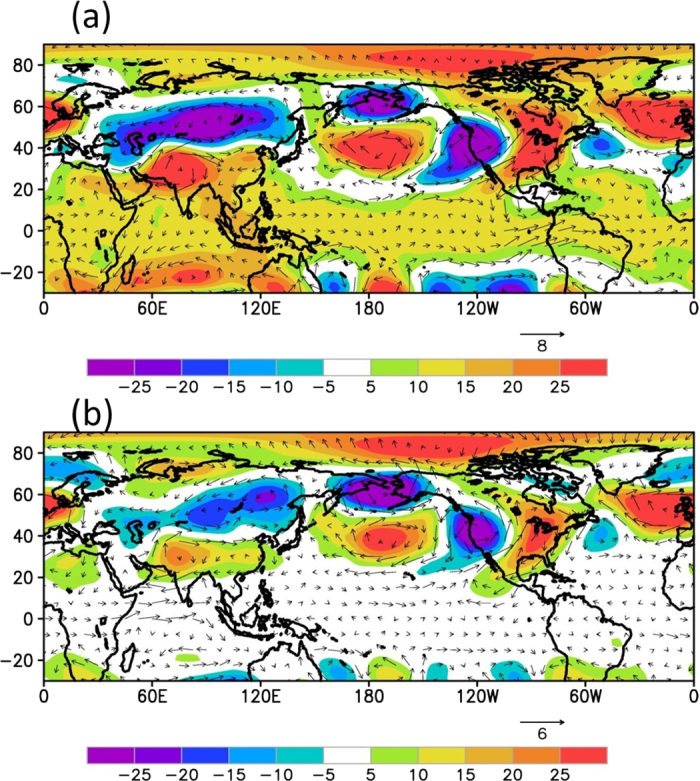
Composite anomalies of geopotential height (gpm) and vector winds at (**a**) 200 hPa level and (**b**) 500 hPa level during the major heat wave events. The major heat wave events are given in Table S1. The geopotential height in gpm is shown as shaded and the winds are shown in wind barbs. This figure was prepared using the Grads version 2.0.1.oga.1 software (http://www.iges.org/grads).

An analysis was therefore made to understand physical mechanisms for occurrence of heat waves over India. For this purpose, major heat wave events during the summer season were identified using area averaged (22^o^–31^o^N, 70^o^–77^o^E) daily maximum temperatures and satisfying the heat wave criteria consecutively for five days. The 27 major heat wave events thus identified are shown in Table S1. A composite analysis of geopotential height and wind anomalies at 200 hPa and 500 hPa levels was made for the selected major heat wave events. The composite height and wind anomalies at 200 and 500 hPa levels averaged for these 27 events are shown in Fig. 3 a and b respectively.

The composite anomalies suggest presence of positive height anomalies and anomalous anticyclonic flow over northern parts of India at 500 hPa and 200 hPa levels. They also show the presence of quasi-stationary Rossby wave pattern with anomalous cyclonic and anticyclonic flow over northern mid-latitudes. There is an anomalous blocking high over the north Atlantic and adjoining Europe with an anomalous low over central Asia. The anomalous high and associated anticyclonic flow is observed over northern parts of India, the area of predominant heat wave activity. Classical blocking highs have been responsible for numerous major heat waves including the Russian, European and Chicago events^13^. The blocking highs do not allow mixing of cold polar air with warm tropical air, thus cause surface warming. However, the anomalous high pressure over northern parts of India is not associated with the classical blocking, but related to sub-tropical high. This high is generally termed as persistent high^13^. The persistent high and associated anti-cyclonic flow in the middle and upper troposphere cause sinking motion, which leads to surface warming due to adiabatic compression.

**Figure 4:**
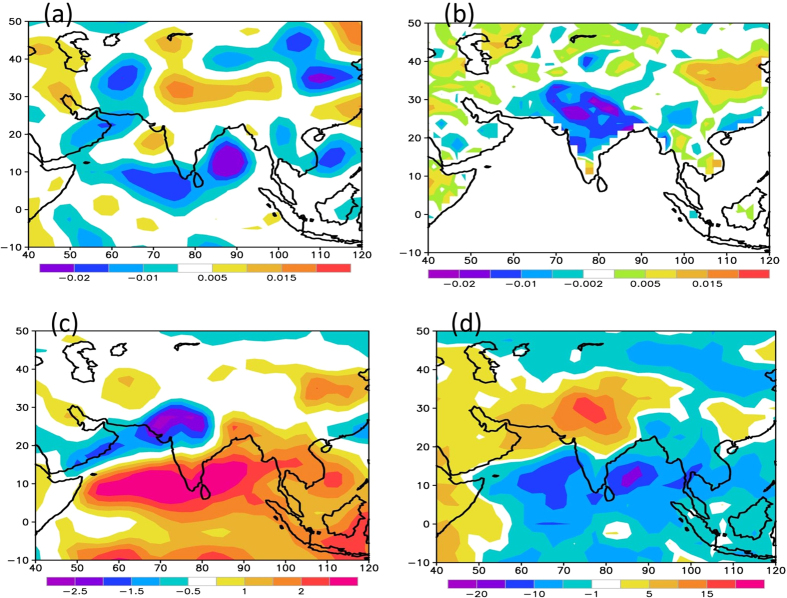
Composite anomalies of (**a**) 500 hPa Omega (Pa/sec) (**b**) Soil Moisture (fraction) (**c**) columnar precipitable water (Kg/m^2^) and (**d**) Outgoing Longwave Radiation (W/m^2^) during the major heat wave events, as given in Table S1. This figure was prepared using the Grads version 2.0.1.oga.1 software (http://www.iges.org/grads).

Figure 4(a) shows vertical velocity at 500 hPa, which clearly suggests sinking motion (with positive values) over the northern parts of the country. These atmospheric conditions are also associated with depleted soil moisture (as shown in Fig. 4b) and reduced precipitable water content over northern parts of the country (Fig. 4c). These conditions are also associated with clear skies with large positive anomalies of outgoing longwave radiation (OLR) (Fig. 4d). Previous studies^20^,^21^,^22^ showed that soil moisture/temperature interactions increase summer temperature variability, resulting in extreme temperatures when soil moisture is low. Reduced soil moisture leads to reduced latent heat transfer into the atmosphere, but enhanced sensible heat transfer inducing a positive feedback between atmospheric heating and further drying of the soil. Dry soil moisture conditions along with persistent high, amplifies the positive feedback and enhances surface warming.

Previous studies^23^,^24^ have documented the influence of major climate anomalies like El Nino/Southern Oscillation (ENSO), North Atlantic Oscillation (NAO) and Pacific Decadal Oscillation (PDO) on the variability of heat waves. In order to understand the physical mechanisms causing the variability of heat waves over India, an analysis was made to examine the coupled mode of variability of heat waves over India with tropical sea surface temperatures. For this purpose, a Canonical Correlation Analysis (CCA) was made using the data of April-May-June (AMJ) tropical SST anomalies and heat wave duration. The CCA was made with the data for the period 1961–2013. Among other statistical methods, CCA has the advantage to select pairs of optimally correlated spatial patterns which may lead to a physical interpretation of the mechanism controlling the climate variability.

**Figure 5:**
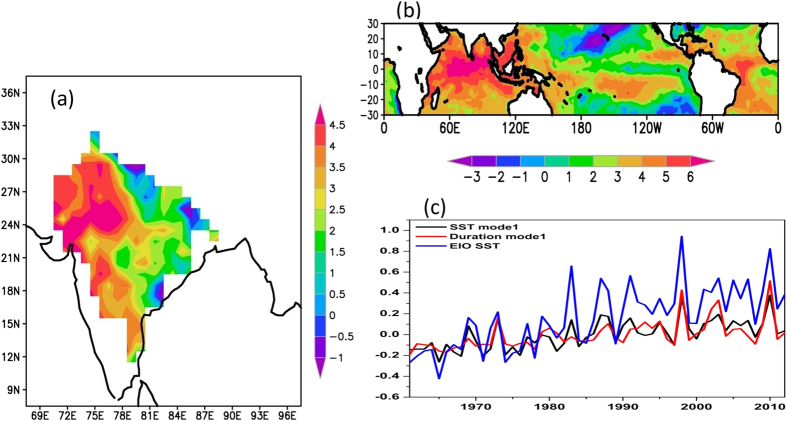
The first canonical mode of the canonical correlation analysis of April-June SST and heat wave duration days for the period 1961–2013. (**a**) Spatial mode of heatwave duration (**b**) spatial mode of SST and (**c**) time series of first mode of SST and heat wave duration. In (**c**), the time series of SST averaged over the equatorial Indian Ocean (EIO) (10°S–10°N, 50°E–100°E) is also shown. This figure was prepared using the Grads version 2.0.1.oga.1 software (http://www.iges.org/grads).

The spatial patterns and CCA time series of the first mode (CCA1) are shown in Fig. 5. The first mode for heat wave duration explains about 31% of variability and that of SST by 26.9%. The first SST spatial mode shows large loading over the tropical Indian Ocean, suggesting a major influence of the tropical Indian Ocean on the variability of heat waves. The first heat wave spatial mode shows large loading over northwest India with opposite phase over northeastern parts of the country. The canonical time series of the first mode (PC1) is also shown in Fig. 5, which shows increasing trends of SST and heat wave duration. The first canonical mode represents increasing trends in heat wave duration over India and also signifies the possible role of the tropical Indian Ocean SST anomalies. The time series of SST anomalies averaged over the equatorial Indian Ocean (10°S–10°N, 50°E–100°E) shown in Fig. 5 also suggests a warming trend. The correlation coefficient between the PC1 of heat wave and the Indian Ocean SST anomalies is 0.78, which explains about 60% variability. Recent studies^25^,^26^ have documented an increased warming of the tropical Indian Ocean during the past half-century. This warming seems to be the largest contributor to the overall trend in the global mean sea surface temperatures^25^. The Indian Ocean warming is attributed both to greenhouse warming and the asymmetry of ENSO teleconnection with the Indian Ocean. Therefore, the present analysis reveals that increasing trends of heat wave duration over the Indian sub-continent are partly influenced by warming of the tropical Indian Ocean. Warmer equatorial Indian Ocean can cause local convection during the April-June season (as seen of negative OLR anomalies in Fig. 4d). The ascending branch due to convection in the equatorial Indian Ocean descends over the northern parts of India (as seen in positive omega values in Fig. 4a and positive OLR anomalies in Fig. 4d). The Latitude-height cross section of omega (pressure vertical velocity) during the heat wave events (not shown) clearly shows the large scale ascending motion over the equatorial Indian ocean and descending motion over the northern parts of the country.

**Figure 6:**
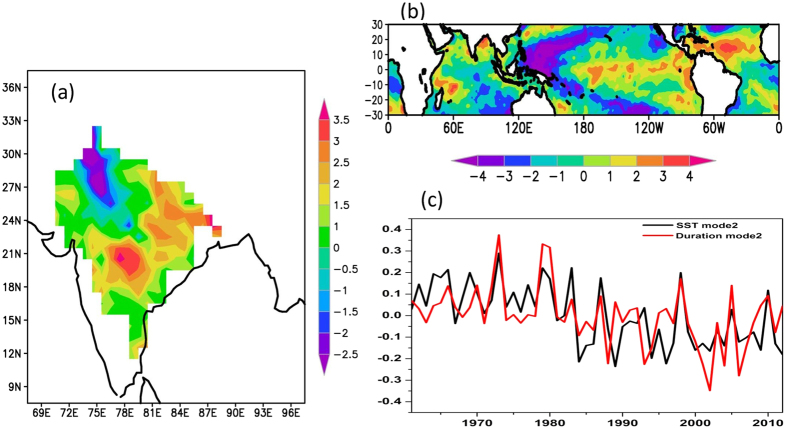
The second canonical mode of the canonical correlation analysis of April-June SST and heat wave duration days for the period 1961–2013. (**a**) Spatial mode of heat wave (**b**) spatial mode of SST and (**c**) time series of second mode of SST and heat wave duration. This figure was prepared using the Grads version 2.0.1.oga.1 software (http://www.iges.org/grads).

The spatial patterns and time series of the second mode are shown in Fig. 6. The spatial pattern of the second mode of heat wave duration shows a dipole pattern (between northwest India and southeast India), which explains 12% of variance. The spatial pattern of the second mode of SST shows more loading from the tropical Pacific region, which suggests the ENSO phenomenon is also a major factor influencing heat wave events over the Indian sub-continent. The time series of frequency, duration and maximum duration (Fig. 3) clearly suggests a link between the El Nino events and heat wave events over India. For example, most of the years with above normal heat wave activity over India (as shown in Fig. 3) are the years following the El Nino events. For example, 1988, 1995, 1998 and 2010 are the years with above normal heat wave activity with concurrent near neutral to weak ENSO conditions in the tropical Pacific. However, we did not find out any significant difference between canonical El Nino events and El Nino Modoki events^34^. The previous study^15^ also revealed that heat wave activity over India increases subsequent to an El Nino year. The third CCA mode (Fig. S3) also suggests large loading from the east equatorial Pacific and a horseshoe pattern of loading resembling of SST anomalies associated with the El Nino events.

## Summary and Discussions

The present study has brought out the salient features of variability of heat waves over India and associated physical mechanisms and influence of coupled climate modes. The past observations of heat waves (1961–2013) clearly suggest a statistically significant increase in frequency, duration and maximum length of duration of heat waves over India. The heat waves are associated with large scale atmospheric anomalies connecting sub-tropical persistent high, quasi-stationary Rossby waves over the mid-latitudes, depleted soil moisture and clear skies. The study has also brought out the possible role of Indian Ocean SST anomalies and the El Nino events on heat wave events over India. The dependence of heat waves on the Indian and Pacific Oceans has large implications. The previous studies^25^,^26^ suggested an increasing trend of SSTs over the tropical Indian Ocean during the recent half-century. This warming trend is likely to continue in future climate in view of increasing greenhouse gases. Similarly, the study by Cai *et al.*^27^ suggested that mean climate of the tropical Pacific is expected to change in future due to greenhouse warming. The consequences of these changes in the mean state include an increased frequency of extreme El Nino events. With warming of the tropical Indian Ocean and increasing frequency of extreme El Nino events, more frequent and long lasting heat wave events are likely over the Indian sub-continent in near future. A separate study on how CMIP5 models project changes in temperature extremes including heat waves over India is therefore desired. A recent study^35^ analyzed climate model temperature output for the period 1950–2100 and documented the global changes in extreme temperatures by using various climate indices. The study suggested significant increase in temperature extremes like warm days over the Indian region during the period 2001–2100. Therefore, there is an urgent need to develop a strategy for forewarning and mitigation efforts to minimize adverse effects of heat waves over the country.

## Methods

### Data

For analysis of heat waves, the high resolution daily temperature gridded data set developed by the India Meteorological Department^28^ has been used. This data set was developed using daily minimum (nighttime) and maximum (daytime) temperature data from 395 synoptic stations spread uniformly over the country. For interpolating station data into regular grids, the modified version of the Shepard’s angular distance weighting algorithm^29^ was used. In order to avoid biases in the gridding, daily temperature anomalies were used instead of absolute values. For this purpose, climatological normal of maximum and minimum temperatures for the period 1971–2000 was calculated for each station. The interpolation method requires an understanding of the spatial correlation structure of the station data. Therefore, inter-station correlations were calculated to determine the distances over which observed temperature anomalies are related. Before interpolation, station data were subjected to preliminary quality controls like removing outliers and ensuring homogeneity. Also, it was ensured that all the stations have the same data length to avoid errors in gridded data due to inhomogeneity in station density. Initially, the gridded data set was developed for the period 1969–2005, which was later updated for the period 1961–2013 using the same set of station data. For the present analysis, the recent 53 years of gridded data (1961–2013) have been considered. More details of the temperature data set are available in Srivastava *et al.*^28^.

For analyzing the physical relationships of heat waves with atmospheric and ocean parameters, we have used different climate data sets. For sea surface temperature, the HADISST data set^30^ provided by the UK Met office Hadley Centre was used. Various atmospheric parameters like geopotential height, vertical velocity, winds, soil moisture and precipitable water were taken from the NCEP-NCAR reanalysis^31^. To examine sensitivity of results on the climate data, we have repeated the analysis using the NCEP ERSST data^36^ for SST and ECMWF ERA data^37^ for atmospheric parameters. Since there were no appreciable differences in the results, we have considered the results based on the HADISST and NCEP-NCAR reanalysis data sets for discussions.

### Heat wave Indices

There is no uniform definition or criterion of heat waves. There is a large diversity of heat waves with more than 15 indices^32^. In India, the India Meteorological Department (IMD) uses station data for monitoring heat waves and uses maximum temperature data and threshold of anomalies^10^.

In the present study, two different indices were considered for identifying heat waves. The first one is the 90^th^ percentile threshold of maximum temperature (T_max_) based on a 5-day window (Tmax_90_). For accounting the seasonal cycle, different threshold percentile value for each day was considered. The 5-day moving window was considered to account for temporal dependence while producing a reasonable sample size to calculate a realistic percentile value. These thresholds are calculated for each time period and grid box separately.

The second index considered is the Excessive Heat Factor^32^,^33^. This index is based on two excessive heat indices, namely Excess Heat and Heat Stress. The Excess heat represents unusually high heat arising from a daytime temperature that is not sufficiently discharged overnight due to unusually high overnight temperatures. The Daily Mean Temperature (DMT) averaged over a three-day period is compared against a climate reference value to characterize this index.

The Excess Heat Index is denoted by


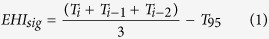


where T_95_ is the 95^th^ percentile of DMT (T_i_) for the climate reference period of 1961–1990. Daily mean temperature is the average of maximum and minium temperatures as defined by


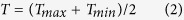


The units of EHI_sig_ are °C.

The heat stress which arises from a period where temperature is warmer, on average, than the recent past. Maximum and subsequent minimum temperatures averaged over a three-day period and the previous 30 days are compared to characterize the heat stress. This is expressed as a short term (acclimatization) temperature anomaly.

The heat stress is defined as


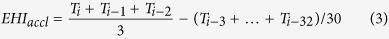


where T_i_ is the DMT on day i. In effect, EHI_accl_ is an anomaly of three-day DMT with respect to the previous 30 days. The units of EHI_accl_ are °C.

Excess Heat Factor (EHF) is the combined effect of Excess Heat and Heat Stress calculated as an index, which provides a comparative measure of frequency, duration and spatial distribution of a heat wave event. Positive values of EHF suggest heat wave conditions.

The Excess Heat Factor (EHF) is defined as follows:


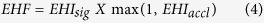


The units of EHF are °C^2^.

There are many advantages of using EHF, which is the most appealing index for heat waves^32^,^33^. For EHF, we consider both T_min_ and T_max_ within the same index, thus considering the moisture effect. Another advantage is that the conditions leading up to a given day (from two days before) are also considered. This can amplify or dampen heat wave amplitude. The given day is also compared an extreme threshold (95^th^ percentile) to determine its climatological anomaly and to the mean of the preceding month to determine the anomaly against recent conditions. This takes into account of accimatization to the heat wave.

Using the above mentioned two indices (Tmax_90_ and EHF), heat wave is defined if the conditions are observed consecutively for three days during the season of April to June. The heat wave statistics was calculated for spatial distribution (at each grid point) as well as averaged over a large area.

Further, the trends were calculated for the following attributes of heat wave.

1. Average frequency of heat wave events per season.
2. Total duration (days) of heat wave events per season.
3. Maximum length (days) of heat waves per season.

The Mann Kendall test for trends of the above parameter was done and trends are indicated as per decade. In the main section, the results based on EHF are discussed due to its higher relevance. The results based on Tmax_90_ are shown in the supplementary document.

## Additional Information

**How to cite this article**: Rohini, P. *et al.* On the Variability and Increasing Trends of Heat Waves over India. *Sci. Rep.* **6**, 26153; doi: 10.1038/srep26153 (2016).

## Supplementary Material

Supplementary Information
