# Supplementary material for: On the Variability and Increasing Trends of Heat Waves over India
Source: Sci Rep. 2016 May 19;6:26153. doi: 10.1038/srep26153 (PMC4872222; doi:10.1038/srep26153)
Supplement: Supplementary Information [file srep26153-s1.doc]

Supplementary Material for the paper “On the variability and increasing trends of heat waves over India”

P.Rohini^1^, M. Rajeevan^1*^ and A.K. Srivastava^2^

1. Indian Institute of Tropical Meteorology, Pune, 411 008, India

2. India Meteorological Department, Pune, 411 005, India


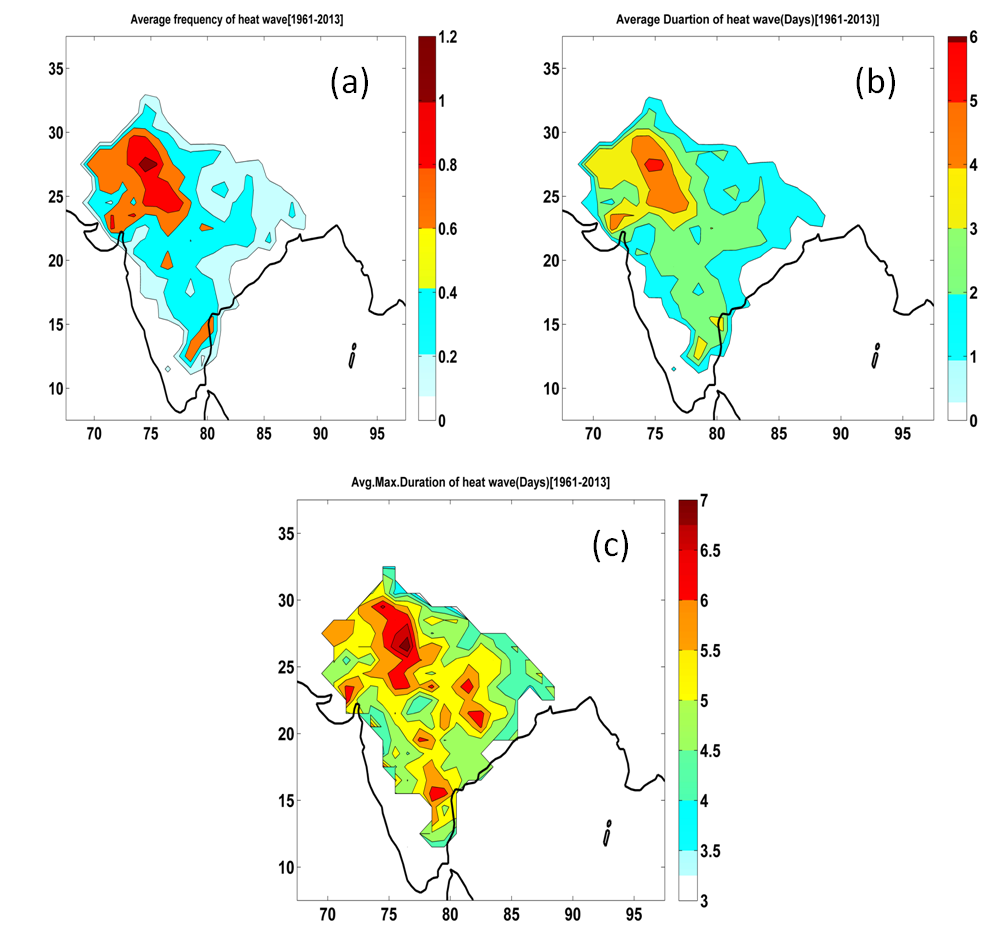
* Corresponding Author

Fig S-1 Climatological features of heat waves over India using the EHF index during the period 1961-2013 a) mean frequency b) total duration (days) and c) maximum length of duration of heat waves. This figure was prepared using the Matlab version R2012a software ([http://in.mathworks.com](http://in.mathworks.com/)).


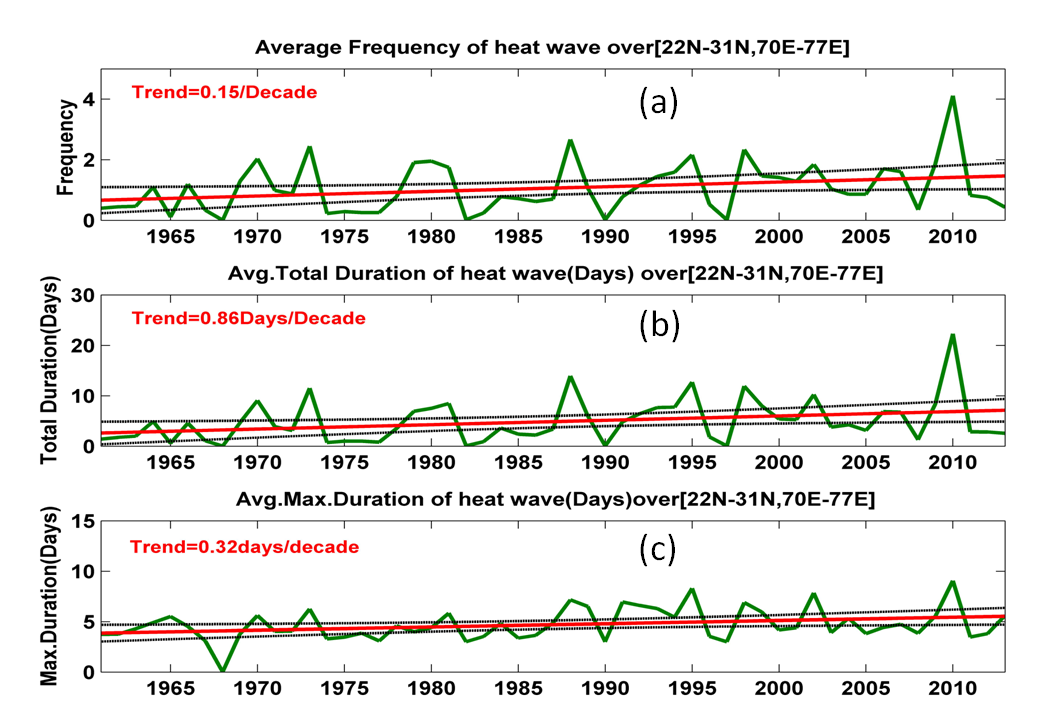
Fig S2: Time series of a) frequency b) total duration (days) and c) Maximum length of duration of heat waves over northwest India (22-31N, 70-77E) during the period 1961-2013 based on Tmax­_90_ index for heat wave. The linear trend line(Red) and the 95% confidence limits(black) are also shown.


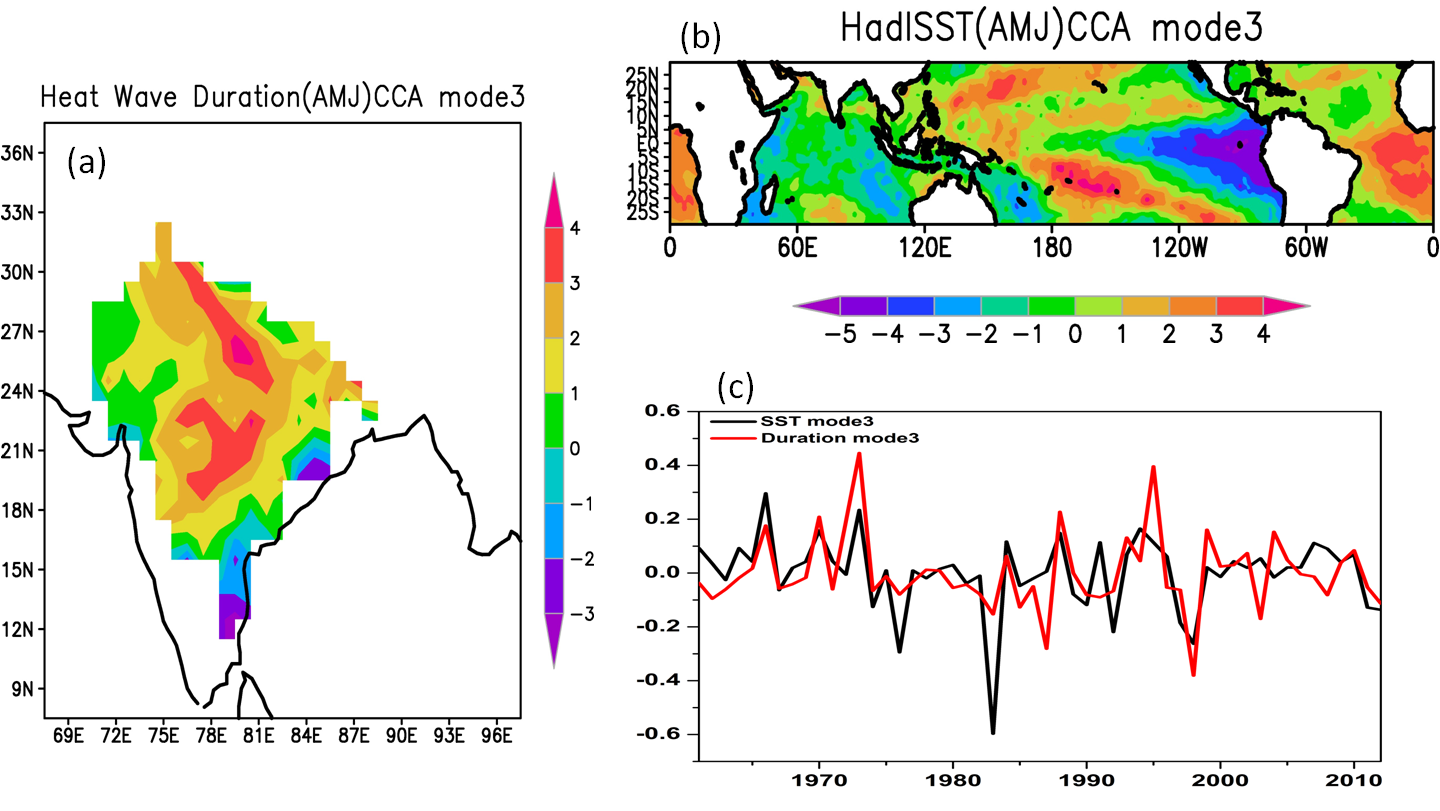


Fig S3: The third canonical mode of the canonical correlation analysis of April-June SST and heat wave duration days for the period 1961-2013. a) spatial mode of SST b) spatial mode of heat wave duration and c) time series of third mode of SST and heat wave duration. This figure was prepared using the Grads version 2.0.1.oga.1 software (http://www.iges.org/grads).

Table: S-1

List of major heat wave events considered for the composites

| **Year** | **Heat Wave Spells** |
| --- | --- |
| **1967**  **1970**  **1971**  **1973**  **1980**  **1981**  **1987**  **1988**  **1989**  **1991**  **1992**  **1993**  **1994**  **1995**  **1998**  **1999**  **2002**  **2009**  **2010**  **2012** | **23-29Jun**  **23-27Apr**  **8-12Apr**  **27Apr-2may**  **1-6Jun**  **15-23Jun**  **18-23Apr,26-30Jun**  **13-18Apr,6-14May**  **15-23May**  **1-6Jun**  **11-21Jun**  **8-12Jun**  **28May-2Jun**  **7-11May,30May-6Jun**  **15-28May**  **6-12Apr,30Apr-4May**  **1-8May**  **27Apr-2May**  **5-11Apr, 13-19Apr, 12-17May, 19-26May**  **2-6Apr** |
